# Complete Genome Sequences of Two *Bordetella hinzii* Strains Isolated from Humans

**DOI:** 10.1128/genomeA.00965-15

**Published:** 2015-08-27

**Authors:** Michael R. Weigand, Shankar Changayil, Yasvanth Kulasekarapandian, M. Lucia Tondella

**Affiliations:** Centers for Disease Control and Prevention, Atlanta, Georgia, USA

## Abstract

*Bordetella hinzii* is primarily recovered from poultry but can also colonize mammalian hosts and immunocompromised humans. Here, we report the first complete genome sequences of *B. hinzii* in two isolates recovered from humans. The availability of these sequences will hopefully aid in identifying host-specific determinants variably present within this species.

## GENOME ANNOUNCEMENT

*Bordetella* species are associated with a variety of hosts, in which they are often the etiologic agents of disease. *Bordetella hinzii* frequently colonizes poultry and causes respiratory disease in turkeys (1). However, *B. hinzii* can also opportunistically infect immunocompromised humans, leading to conditions such as respiratory disease, septicemia, and cholangitis (2–5). Rodents have been proposed to serve as reservoirs for *B. hinzii* (6, 7), suggesting that this species may be an emerging zoonotic pathogen. Therefore, comparative genomics of *B. hinzii* isolated from different sources is needed to elucidate any virulence mechanisms defining the host range in this species. Here, we report the complete genome sequences of two clinically derived *B. hinzii* strains from the culture collection at the Centers for Disease Control and Prevention. *B. hinzii* F582, a clone of ATCC 51730, was isolated from a blood sample from a male AIDS patient (8), and *B. hinzii* H568 was isolated in 2010 from a sputum sample from a male in Arkansas. Although at least eight draft genomes for *B. hinzii* exist in public databases (9, 10), including that of ATCC 51730, these are the first closed genome sequences for this species.

Genome sequencing was performed using a combination of the PacBio RSII (Pacific Biosciences, Menlo Park, CA) and HiSeq PE-100 (Illumina, Hayward, CA) platforms. Genomic DNA was isolated with the Gentra Puregene yeast/bacteria kit (Qiagen, Valencia, CA). Libraries were prepared for PacBio sequencing using the SMRTbell template prep kit 1.0 and polymerase binding kit P4, while HiSeq libraries were prepared using the NEBNext Ultra library prep kit (New England BioLabs, Ipswich, MA). PacBio sequencing reads were filtered and assembled *de novo* using the Hierarchical Genome Assembly Process algorithm (HGAP) version 3. The initial assemblies were improved by unambiguously mapping reads with BLASR (version 1) at 482× and 184× coverage for F582 and H568, respectively. The resulting consensus sequences were determined with Quiver (version 1) and manually checked for circularity. The assemblies were validated by comparison to restriction digest optical maps generated with the Argus platform (OpGen, Gaithersburg, MA) using MapSolver (version 2.1.1). Finally, the genome sequences were further polished by mapping HiSeq reads at 184× and 81× coverage for F582 and H568, respectively. Raw reads were first quality trimmed, clipped of adapter contamination, and filtered using cutadapt (version 1.9) (11). The reads were mapped against the HGAP assembly using the basic variant detection module in CLC Genomics Workbench (version 7.5) to identify errors, which were then corrected using a custom Perl script.

The average G+C content of both genomes is 67.1%, and the genome sizes were 4,972,977 and 4,859,884 bp for F582 and H568, respectively. Genome annotation with Prokka (version 1.11) (12) identified 4,559 protein-coding genes and 64 tRNAs in F582 and 4,467 protein-coding genes and 66 tRNAs in H568. Both genomes contain three rRNA operons. A comparison of the two genomes by read mapping in CLC Genomics Workbench or *k*-mer alignment with kSNP (version 3.0) (13) revealed 8,926 to 9,688 core single-nucleotide polymorphisms (SNPs), depending on the method of identification. The two sequences also include several strain-specific genomic islands, some of which include unique prophage insertions.

### Nucleotide sequence accession numbers.

The complete genome sequences have been deposited at DDBJ/EMBL/GenBank under the accession numbers CP012076 and CP012077 for *B. hinzii* strains F582 and H568, respectively. The versions described in this paper are the first versions.
