# Correction for Weigand et al., Complete Genome Sequences of Two *Bordetella hinzii* Strains Isolated from Humans

**DOI:** 10.1128/genomeA.01639-15

**Published:** 2016-01-14

**Authors:** Michael R. Weigand, Shankar Changayil, Yasvanth Kulasekarapandian, Dhwani Batra, Vladimir Loparev, Phalasy Juieng, Lori Rowe, Mili Sheth, Jamie K. Davis, M. Lucia Tondella

**Affiliations:** Centers for Disease Control and Prevention, Atlanta, Georgia, USA

## AUTHOR CORRECTION

Volume 3, no. 4, e00965-15, 2015. Page 1: The byline and affiliation line should read as given above.

